# Editorial for the Special Issue on Microfluidics for Soft Matter and Mechanobiology

**DOI:** 10.3390/mi11040372

**Published:** 2020-04-02

**Authors:** Sung Sik Lee

**Affiliations:** 1 Scientific Center for Optical and Electron Microscopy, ETH Zurich, Zurich, CH 8093, Switzerland; leesu@ethz.ch; 2 Institute of Biochemistry, ETH Zurich, Zurich, CH 8093, Switzerland

Microfluidics has proven to be a useful platform to understand the material properties and technical applications of soft matter, including emulsions, polymer solutions, hydrogels, and cellulose papers. The study of the characteristics of soft matter, like viscoelasticity, non-Newtonian fluid mechanics, and deformation, has greatly benefitted from using microfluidics to accurately control conditions in time and space. Microfluidics has also served as a useful platform to study biological cell and tissues systems, including mechanobiology. Using microfluidics, external mechanical stress is regulated in physiologically-relevant systems for studying cells, tissues and organisms to understand how mechanical cues are sensed and transduced into biochemical and electrical signals that influence mechano-transduction. Furthermore, the characteristics of soft matter are exploited when combined with microfluidic platforms to mimic *in-vivo* microenvironments like an extracellular matrix to directly test the influence of mechanical cues such as softness and elasticity. In addition, microfluidics platforms enable us to measure the mechanical properties of cells by establishing defined flow or confined microstructures through viscoelastic particles/cells focusing and droplet microfluidics. Finally, the flexible microdevices have become widely employed.

In this Special Issue, we highlight recent progress in microfluidics with research papers and review articles that focus on novel methodological developments and applications of microfluidics devices for soft matter and mechanobiology. It contains ten research papers and two review articles on the following aspects of microfluidic application regarding soft matter and mechanobiology: (1) droplet generation and its application (2) viscoelasticity-based handling of particles/cells (3) paper-based assays (4) flexible devices (5) *in-vivo* microenvironment mimics and (6) mechanobiology research.

1. Droplet generation and its application: Sánchez et al. [1] reviewed recent advances in droplet-based microfluidic technologies for biochemistry and molecular biology. Zeng et al. [2] presented a simple way to predict droplet generation speed in microfluidics device. Chung et al. [3] utilized multilayer parallelized microfluidics geometry for the scalable production of microspheres. Nasser et al. [4] demonstrated a PMMA-based microfluidics device for cost-effective PCR applications. Kim et al. [5] made homogenous amino-functionalized hydrogel microbeads for on-bead bioassay.
2. Viscoelasticity-based handling of particles/cells: Cho et al. [6] found the effect of ionic strength on lateral particle migration in shear-thinning fluids. Lim et al. [7] and Nam et al. [8] utilized viscoelasticity to enrich circulating tumor cell [7] and *C. albicans* [8] in sheathless flow conditions.
3. Paper-based assays: Kim et al. [9] reviewed recent advances of fluidic manipulation technologies in paper-based microfluidic assays.
4. Flexible devices: Lee et al. [10] demonstrated direct patterning of a carbon nanotube thin layer on a stretchable substrate.
5. Mimic of *in-vivo* microenvironments: Yue et al. [11] engineered vascular-like microstructures by microfluidic construction of multilayered hydrogel microtubes.
6. Mechanobiology research: Feng et al. [12] discussed the recent advance and the need for future toolbox development in mechanobiology research of intracellular organelles.

We wish to thank all authors who submitted their manuscripts to this Special Issue. We would also like to acknowledge all the reviewers for dedicating their time to provide careful and timely reviews to ensure the quality of this Special Issue.
